# Supplementary material for: Mobility changes following COVID-19 stay-at-home policies varied by socioeconomic measures: An observational study in Ontario, Canada
Source: PLOS Glob Public Health. 2024 Nov 26;4(11):e0002926. doi: 10.1371/journal.pgph.0002926 (PMC11594434; doi:10.1371/journal.pgph.0002926)
Supplement: S1 Text — (DOCX) [file pgph.0002926.s002.docx]

**Supporting Information**

**S1 Text. Definition of the mobility metric: % of devices that went outside primary location**

The mobility metrics were generated by BlueDot (BlueDot Inc., Toronto, Canada) for the Ontario Ministry of Health using anonymized, population-aggregated location data from mobile devices at 30 minute intervals sourced by BlueDot, from Veraset (Veraset, San Francisco, United States), a data-as-a-service vendor.

The primary location of a given device was first measured on a daily basis using 600 squared meter grid where the device spent most of its time (often referred to as dwell time) during the night hours (12:00am-9:00am Eastern Time). The daily measure of all mobility metric per unique device was then assigned a census tract and averaged weekly to generate a spatially and temporally aggregated metric of the % of devices that left the primary location for at least 30 minutes on a given day. The range is from 0% to 100%. We identified the time index of our weekly data by its first day of the epidemiological week (Sunday-Saturday). The same procedure was used for corresponding weeks in 2019 to construct a weekly reference of pre-pandemic mobility for each census tract.
