# Supplementary material for: Mobility changes following COVID-19 stay-at-home policies varied by socioeconomic measures: An observational study in Ontario, Canada
Source: PLOS Glob Public Health. 2024 Nov 26;4(11):e0002926. doi: 10.1371/journal.pgph.0002926 (PMC11594434; doi:10.1371/journal.pgph.0002926)
Supplement: S2 Text — (DOCX) [file pgph.0002926.s003.docx]

**S2 Text. Source of COVID-19 person-level data**

In Ontario, the reported SARS-CoV-2 cases were obtained from the provincial Case and Contact Management data as part of the Public Health Ontario Integrated Public Health Information System via the Ontario COVID-19 Modelling Consensus Table [1]. The surveillance data included geographic information on census tract and public health unit. We used the person-level information on census tract to aggregate the data to the census-tract level. The data were made available by the Ontario Ministry of Health to the Ontario Modelling Consensus Table on a daily basis.

**References**

1. Hillmer MP, Feng P, McLaughlin JR, Murty VK, Sander B, Greenberg A, et al. Ontario’s COVID-19 Modelling Consensus Table: mobilizing scientific expertise to support pandemic response. Canadian Journal of Public Health. 2021;112(5):799-806.
