# Supplementary material for: Mobility changes following COVID-19 stay-at-home policies varied by socioeconomic measures: An observational study in Ontario, Canada
Source: PLOS Glob Public Health. 2024 Nov 26;4(11):e0002926. doi: 10.1371/journal.pgph.0002926 (PMC11594434; doi:10.1371/journal.pgph.0002926)
Supplement: S3 Text — (PDF) [file pgph.0002926.s004.pdf]

## S3 Text. Model details for difference-in-differences analysis with mixed-effect models for the $2^{nd}$ restriction policy

Let $y_{i,t}$ be the observation (i.e. `Adjusted Mobility`) in census track (CT) $i$ and at week $t$, $t = 1, 2, \ldots, 6$, one of the six weeks during the study period. Let $\texttt{Week}_{i,t} \in \{1, 2, 3, 4, 5, 6\}$ be a categorical time variable, $\texttt{Quintile}_i \in \{1, 2, 3, 4, 5\}$ be a socioeconomic quintile variable, $\texttt{Group}_{i,t}$ be a time-invariant binary indicator denoting whether CT $i$ was in the treatment/intervention group (i.e. Toronto and Peel public health units), $\texttt{Restriction}_{i,t}$ be a binary indicator denoting whether CT $i$ was under restriction in week $t$. Let $j(i)$ denote the public health unit (PHU) of the CT $i$. We have that $i = 1, 2, \ldots, 1240$ and $j = 1, 2, 3, 4, 5$ denoting 1240 CTs within 5 PHUs in this study.

For **Model 1**, we show it as equation 1:

$$
\begin{aligned}
y_{i,t} =& \beta_0 + \gamma_t \texttt{Week}_{i,t} + \beta_1 \texttt{Group}_{i,t} + \beta_2 \texttt{Restriction}_{i,t} \\
& + \phi_i + \alpha_{j(i)} + \epsilon_{i,t}
\end{aligned} \tag{1}
$$

where week 1 (i.e. $t = 1$) is the baseline level. $\beta_0$ is the fixed intercept, $\gamma_t$ is the fixed time effect, $\beta_1$ is the fixed effect of group variable, $\beta_2$ is the fixed effect of restriction, $\phi_i \sim N(0, \theta^2)$ is the CT-level random intercept, $\alpha_{j(i)} \sim N(0, \tau^2)$ is the PHU-level random intercept, and $\epsilon_{i,t} \sim iid\, N(o, \sigma^2)$. The expression $iid$ stands for independent and identically distributed.

For **Model 2A** and **Model 2B**, we show them as equation 2:

$$
\begin{aligned}
y_{i,t} =& \beta_0 + \gamma_t \texttt{Week}_{i,t} + \beta_1 \texttt{Group}_{i,t} + \gamma_{q(i)} \texttt{Quintile}_i \\
& + \beta_2 \texttt{Restriction}_{i,t} + \delta_{tq(i,t)} \texttt{Week}_{i,t} \cdot \texttt{Quintile}_i \\
& + \delta_{q(i)} \texttt{Group}_{i,t} \cdot \texttt{Quintile}_i + \eta_{q(i)} \texttt{Restriction}_{i,t} \cdot \texttt{Quintile}_i \\
& + \phi_i + \alpha_{j(i)} + \epsilon_{i,t}
\end{aligned} \tag{2}
$$

where $q \equiv q(i) \in \{1, 2, 3, 4, 5\}$ is a socioeconomic quintile that $i^{\text{th}}$ CT belongs to and quintile 1 (i.e. $q = 1$) is the baseline level. $\gamma_{q(i)}$ is the fixed effect of socioeconomic quintile, $\delta_{tq(i,t)}$ is the interaction effect of time variable and socioeconomic quintile, $\delta_{q(i)}$ is the interaction effect of group variable and socioeconomic quintile, and $\eta_{q(i)}$ is the effect modification by socioeconomic quintile on the restriction effect. In **Model 2A**, `Quintile` is the income quintile of CT $i$. In **Model 2B**, `Quintile` is the essential worker quintile of CT $i$.

The random intercept, applied at the level of the census tract, captured spatial autocorrelation of the repeated measures within census tract over time [1]. The use of the random intercept at the level of the public health unit also captured some degree of spatial autocorrelation among census tracts within a given public health unit [1]. The public health unit is the geographic level that we conceptualized as most important since policy-decisions were made and implemented at the level of the public health unit, and as such we anticipated

important correlations within the same public health unit. Due to the above rationale, and considering the model complexity and to avoid overparameterization, and potential identifiability issues, we did not use a specialized correlation structure

# References


1. Bertrand M, Duflo E, Mullainathan S. How much should we trust differences-in-differences estimates? The Quarterly journal of economics. 2004;119(1):249-75.
