# Supplementary material for: Mobility changes following COVID-19 stay-at-home policies varied by socioeconomic measures: An observational study in Ontario, Canada
Source: PLOS Glob Public Health. 2024 Nov 26;4(11):e0002926. doi: 10.1371/journal.pgph.0002926 (PMC11594434; doi:10.1371/journal.pgph.0002926)
Supplement: S4 Text — (DOCX) [file pgph.0002926.s005.docx]

**S4 Text. Sensitivity analysis comparing the results (of the descriptive and the difference-in-differences analysis) between essential workers excluding health care workers and essential workers including health care workers.**

1. **Sensitivity analysis for Objective 1: Descriptive analysis - mobility change following the first restriction by comparing Table 1**

**Table 1. Mobility metric of pre-restriction^a^ and post-restriction^b^ periods for the first restriction in Greater Toronto Area^c^ stratified by neighborhood-level^d^ socioeconomic measures.**

**Original version excluding health care workers**

|  | Mobility^e^ | | | | | |
| --- | --- | --- | --- | --- | --- | --- |
|  | Pre-restriction | | Post-restriction | | Mobility change^f^ | |
| % Essential worker^g^ Quintiles^h^ | Crude^i^ | Adjusted^j^ | Crude | Adjusted | Crude | Adjusted |
| Q1 (lowest %) | 78.5 | 2.1 | 39.0 | -42.3 | -39.5 | -44.5 |
| Q2 | 79.1 | 2.8 | 40.9 | -40.6 | -38.2 | -43.4 |
| Q3 | 78.3 | 2.2 | 43.1 | -37.5 | -35.2 | -39.7 |
| Q4 | 77.6 | 2.6 | 42.7 | -37.4 | -34.9 | -40.0 |
| Q5 (highest %) | 75.3 | 1.6 | 42.6 | -36.1 | -32.7 | -37.6 |

**Essential workers including health care workers**

|  | Mobility | | | | | |
| --- | --- | --- | --- | --- | --- | --- |
|  | Pre-restriction | | Post-restriction | | Mobility change | |
| % Essential worker Quintiles | Crude | Adjusted | Crude | Adjusted | Crude | Adjusted |
| Q1 (lowest %) | 78.3 | 2 | 38.8 | -42.5 | -39.4 | -44.5 |
| Q2 | 79.3 | 3.1 | 41.1 | -40.2 | -38.2 | -43.3 |
| Q3 | 78.6 | 2 | 43.3 | -37.8 | -35.3 | -39.8 |
| Q4 | 77.2 | 2.7 | 42.5 | -37.2 | -34.7 | -39.9 |
| Q5 (highest %) | 75.4 | 1.6 | 42.6 | -36.1 | -32.8 | -37.6 |

^a^Pre-restriction = three weeks before restriction implementation, and excluded the week of implementation (i.e. February 23 to March 8, 2020 for the first restriction);

^b^Post-restriction = three weeks after restriction implementation, and excluded the week of implementation (i.e. March 22, 2020 to April 5, 2020 for the first restriction);

^c^Greater Toronto Area comprised of five public health units (Toronto, Peel, Halton, York, and Durham);

^d^Neighborhood-level variables are at the level of census tract;

^e^Mobility = average % of devices that went outside “home” location;

^f^Mobility change = the post-restriction mobility metric minus the pre-restriction mobility metric;

^g^% Essential worker = proportion of the working population engaged in essential services. Essential services include: trades, transport, and equipment operation; sales and services; manufacturing and utilities; and resources, agriculture, and production.

^h^Quintile (Q) was calculated across five public health units, weighted by neighborhood-level population in terms of the socioeconomic variables;

^i^Crude = mobility metric in 2020;

^j^Adjusted = crude mobility in 2020 minus crude mobility in 2019.

1. **Sensitivity analysis for Objective 1: Descriptive analysis - mobility change following the second restriction by comparing Table 2**

**Table 2. Mobility metric of pre-restriction^a^ and post-restriction^b^ periods for the second restriction in two public health units (Toronto, Peel) stratified by neighborhood-level^c^ socioeconomic measures.**

**Original version excluding health care workers**

|  | Mobility^d^ | | | | | |
| --- | --- | --- | --- | --- | --- | --- |
|  | Pre-restriction | | Post-restriction | | Mobility change^e^ | |
| % Essential worker^f^ Quintiles^g^ | Crude^h^ | Adjusted^i^ | Crude | Adjusted | Crude | Adjusted |
| Q1 (lowest %) | 60.2 | -19.3 | 55.9 | -21.6 | -4.2 | -2.3 |
| Q2 | 61.5 | -17.7 | 58.7 | -18.0 | -2.7 | -0.3 |
| Q3 | 63.0 | -15.8 | 59.3 | -17.7 | -3.7 | -1.9 |
| Q4 | 62.3 | -15.6 | 60.2 | -15.7 | -2.1 | -0.1 |
| Q5 (highest %) | 62.0 | -14.2 | 60.4 | -13.9 | -1.6 | 0.3 |

**Essential workers including health care workers**

|  | Mobility | | | | | |
| --- | --- | --- | --- | --- | --- | --- |
|  | Pre-restriction | | Post-restriction | | Mobility change | |
| % Essential worker Quintiles | Crude | Adjusted | Crude | Adjusted | Crude | Adjusted |
| Q1 (lowest %) | 59.8 | -19.4 | 55.6 | -21.8 | -4.3 | -2.3 |
| Q2 | 61.9 | -17.3 | 58.9 | -18.1 | -2.9 | -0.8 |
| Q3 | 63.6 | -15.6 | 59.8 | -17.4 | -3.8 | -1.8 |
| Q4 | 62.2 | -15.7 | 60.3 | -15.5 | -1.9 | 0.1 |
| Q5 (highest %) | 61.9 | -14.3 | 60.2 | -14 | -1.7 | 0.3 |

^a^Pre-restriction = three weeks before restriction implementation, and excluded the week of implementation (i.e. February 23 to March 8, 2020 for the first restriction);

^b^Post-restriction = three weeks after restriction implementation, and excluded the week of implementation (i.e. March 22, 2020 to April 5, 2020 for the first restriction);

^c^Neighborhood-level variables are at the level of census tract;

^d^Mobility = average % of devices that went outside “home” location;

^e^Mobility change = the post-restriction mobility metric minus the pre-restriction mobility metric;

^f^% Essential worker = proportion of the working population engaged in essential services. Essential services include: trades, transport, and equipment operation; sales and services; manufacturing and utilities; and resources, agriculture, and production.

^g^Quintile (Q) was calculated across five public health units, weighted by neighborhood-level population in terms of the socioeconomic variables;

^h^Crude = mobility metric in 2020;

^i^Adjusted = crude mobility in 2020 minus crude mobility in 2019.

1. **Sensitivity analysis for Objective 2: Difference-in-differences analysis - mobility change following the second restriction by comparing Fig 3 and S6 Table C**

**Fig 3. Adjusted mobility change following the second restriction by neighborhood-level socioeconomic measures in the Greater Toronto Area.** The figure shows the estimates of the adjusted mobility following the second set of restrictions by essential worker quintiles. The error bars represent the 95% confidence interval. The Greater Toronto Area comprised of five public health units (Toronto, Peel, Halton, York, and Durham). Essential services include: trades, transport, and equipment operation; sales and services; manufacturing and utilities; and resources, agriculture, and production. Neighborhood level is defined at the level of the census
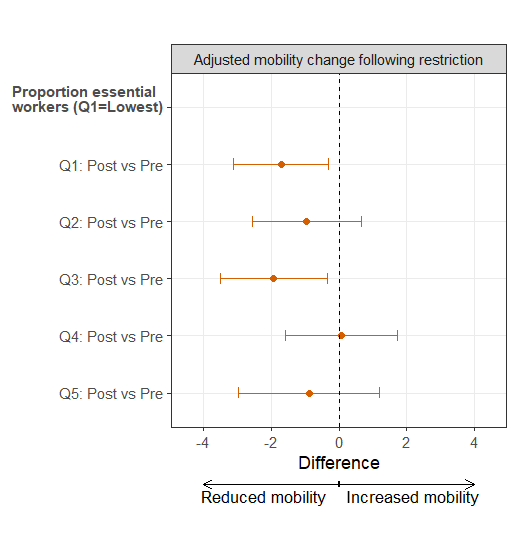
tract. Quintiles (Q) are weighted by neighborhood-level population.

**Original version excluding health care workers**

**Essential workers including health care workers**


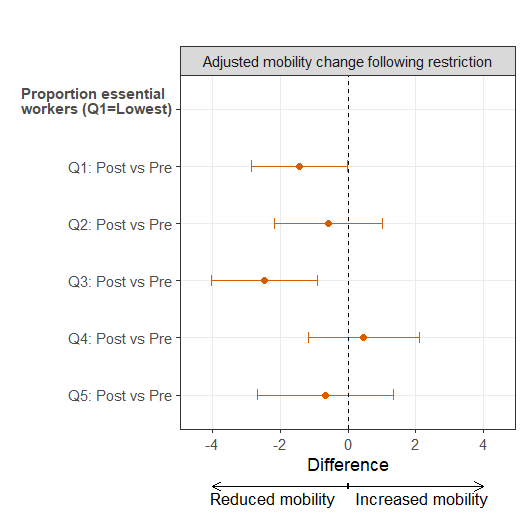


**S6 Table C. Difference-in-differences analysis of the second restriction with mixed-effect modeling in Greater Toronto Area^a^ by area-level^b^ socioeconomic measures.**

**Original version excluding health care workers**

| Quintiles (Q)^c^ | Adjusted mobility^d^ change | 95% CI^e^ |
| --- | --- | --- |
| % Essential workers^f^ |  |  |
| 1 (smallest %) | -1.43 | (-2.85; -0.01) |
| 2 | -0.58 | (-2.19; 1.03) |
| 3 | -2.48 | (-4.05; -0.90) |
| 4 | 0.47 | (-1.17; 2.11) |
| 5 (largest %) | -0.67 | (-2.68; 1.35) |

**Essential workers including health care workers**

| Quintiles (Q) | Adjusted mobility change | 95% CI |
| --- | --- | --- |
| % Essential workers |  |  |
| 1 (smallest %) | -1.71 | (-3.12; -0.31) |
| 2 | -0.95 | (-2.56; 0.66) |
| 3 | -1.93 | (-3.51; -0.35) |
| 4 | 0.07 | (-1.58; 1.72) |
| 5 (largest %) | -0.89 | (-2.99; 1.21) |

^a^Greater Toronto Area comprised of five public health unit (Toronto, Peel, Halton, York, and Durham);

^b^Area-level variables at the level of census tract;

^c^Quintile (Q) was calculated across five public health units, weighted by census tract population size in terms of the socioeconomic variables;

^d^Adjusted mobililty = crude mobility in 2020 minus crude mobility in 2019;

^e^95% CI = 95% confidence interval;

^f^% Essential worker = proportion of the working population engaged in essential services. Essential services include: trades, transport, and equipment operation; sales and services; manufacturing and utilities; and resources, agriculture, and production.

1. **Cross table comparing essential workers excluding health care workers and essential workers including health care workers**

**Essential workers including health care workers**

| Essential worker quintiles | Q1 | Q2 | Q3 | Q4 | Q5 |
| --- | --- | --- | --- | --- | --- |
| Q1 | 17002 | 1260 | 0 | 0 | 0 |
| Q2 | 1400 | 13580 | 1190 | 0 | 0 |
| Q3 | 0 | 1311 | 13860 | 1679 | 0 |
| Q4 | 0 | 0 | 1750 | 14560 | 1050 |
| Q5 | 0 | 0 | 0 | 1176 | 16940 |

**Original version excluding health care workers**
