# Supplementary material for: Mobility changes following COVID-19 stay-at-home policies varied by socioeconomic measures: An observational study in Ontario, Canada
Source: PLOS Glob Public Health. 2024 Nov 26;4(11):e0002926. doi: 10.1371/journal.pgph.0002926 (PMC11594434; doi:10.1371/journal.pgph.0002926)
Supplement: S1 Table — (DOCX) [file pgph.0002926.s006.docx]

**S1 Table. Measures for 1^st^ restriction and 2^nd^ restriction of COVID-19 in Ontario**

| Restriction | Public and social gatherings^a^ | Industry services and retail | Outdoor, recreational and seasonal activities | Care, community, and household services | Academic and teaching institutions |
| --- | --- | --- | --- | --- | --- |
| 1^st^ restriction^b^ [1, 2] | General guidelines   - Limit for outdoor organized public events and social gatherings (excluding those within same household): 5 people - Every person shall stay-at-home except for essential reasons - Physical distancing of at least two metres away from others outside of their direct household   Permitted with requirements   - Funeral service with limit of 10 people^c^ [3] | Open for essential businesses including   - Supply chains - Retail and wholesale of food, pet food and supplies, and household consumer products - Liquor stores - Gas stations and fuel providers - Motor repair - Hardware stores - Pharmaceutical services - Office supplies and services - Safety supply - Restaurants and food facilities^d^ - Hotels and shared rental units - Support and maintenance services - Telecommunications and IT infrastructure/providers - Transportation providers and supporting services - Manufacturers - Agriculture and food production - Construction - Financial activities - Resources - Environmental services - Utilities and community services - Communications industries - Research - Justice sector - Other businesses^e^ - Business regulators and inspectors | All non-essential businesses closed; no essential businesses in this category | Open for essential businesses including   - Child care and day camps - Court services - Government services - Mental health and addictions support services (e.g., Alcoholics Anonymous) permitted to a maximum of 10 people - Provision of social services | Publicly-funded elementary and secondary closed starting March 14, 2020 [4, 5]  Suspension or modification of classes at post-secondary institutions starting mid-March 2020^f^ [6] |
| 2^nd^ restriction [7-9] | General guidelines   - Limit for outdoor organized public events and social gatherings (excluding those within same household): 10 people - Every person shall stay-at-home except for essential reasons - Physical distancing of at least two metres away from others outside of their direct household - Funeral service with limit of 10 people | Retail generally permitted to be open for curbside pick-up or delivery only (in-person retail shopping not permitted) with some exceptions:   - Supermarkets, grocery stores, convenience stores, hardware stores, other retailers selling groceries, beer and wine and liquor stores, pharmacies and safety supply stores permitted to be open for in-person shopping - 50% capacity limits where in-person shopping is permitted - Motor/recreational vehicle sales permitted to be open for in-person shopping by appointment only and other restrictions - Garden centres, plant nurseries: indoor by appointment. Permitted if public remains outdoors or by curbside pick-up or delivery - Outdoor markets, including farmer’s markets and holiday markets, permitted with restrictions   Retail outlets in malls permitted to be open for pick-up or delivery (in-person retail shopping not permitted)  Access to shopping malls for limited purposes, including access to businesses and organizations permitted to be open (e.g., pharmacy, dentist); food court open for take-away; malls may also establish designated pick-up points inside or adjacent to the mall  Subject to general rule for businesses that are open must maintain 2m while standing in line inside and outside | Indoor and outdoor service closed Take out, drive through, and delivery permitted, including alcohol  Facilities for indoor or outdoor sports and recreational fitness activities are closed except for:   - The sole use of high performance athletes including parasport athletes, and specified professional leagues (e.g., NHL, CFL, MLS, NBA) - Specified purposes (e.g. day camps, child care)   Outdoor recreational amenities (e.g., ice rinks, ski hills, snow trails) open with restrictions (e.g., no team sports) | Open for essential businesses including   - Health care and seniors care and social services - Child care centres | Post-secondary institutions open for virtual instruction. In-person instruction limited (e.g. clinical training, trades) and for examinations. In-person cannot exceed 10 persons.   \|  \| \| --- \| |

^a^Gathering limits and general public health and safety guidelines apply to all businesses, events, and activities. Exceptions are detailed in the footnotes.

bClosure of non-essential businesses – with the exception of essential businesses mentioned, all other businesses are closed.

cGathering limit of 5 people is exempted for funerals.

dOnly for delivery or takeaway.

eOther businesses include rental and leasing services, businesses providing mailing and delivery services, laundry service providers, professional services, businesses providing funeral and related goods and services, land registration/real estate agent services and moving services, security services, staffing services, support of safe operations of residences and essential businesses, businesses that provide for health and welfare of animals, child care services for essential workers, businesses providing cheque cashing services.

fDecisions were made independently by each school

**References**

1. Emergency Management and Civil Protection Act - ONTARIO REGULATION 82/20. , (2020).

2. Ontario Extends Emergency Declaration to Stop the Spread of COVID-19: Government of Ontario; 2020 [cited 2022 May 15]. Available from: <https://news.ontario.ca/en/release/56523/ontario-extends-emergency-declaration-to-stop-the-spread-of-covid-19>.

3. Ontario Regulation REG2020.0205, (2020).

4. Statement from Premier Ford, Minister Elliott, and Minister Lecce on the 2019 Novel Coronavirus (COVID-19). : Government of Ontario, Office of the Premier; 2020 [cited 2022 March 20]. Available from: <https://news.ontario.ca/en/statement/56270/statement-from-premier-ford-minister-elliott-and-minister-lecce-on-the-2019-novel-coronavirus-covid-19>.

5. School closures extended to keep students, staff and families safe 2020: Government of Ontario; [cited 2022 May 17]. Available from: <https://news.ontario.ca/en/release/56776/school-closures-extended-to-keep-students-staff-and-families-safe>.

6. Freeman J. GTA universities, colleges suspending classes amid pandemic. CTV News. 2020.

7. COVID-19 restrictions in Grey Zone of the Province of Ontario’s COVID-19 response framework: City of Toronto; 2021 [cited 2021 July 15]. Available from: <https://www.toronto.ca/news/covid-19-restrictions-in-grey-zone-of-the-province-of-ontarios-covid-19-response-framework/>.

8. Appia V. Here's how Ontario's COVID-19 colour codes work: Scarborough Mirror. Available from: <https://www.toronto.com/news/heres-how-ontarios-covid-19-colour-codes-work/article_1a46f69f-ca18-5b81-8689-769dbb167d22.html>?

9. Ontario declares second provincial emergency to address COVID-19 crisis and save lives: Government of Ontario; 2021 [cited 2022 July 15]. Available from: <https://news.ontario.ca/en/release/59922/ontario-declares-second-provincial-emergency-to-address-covid-19-crisis-and-save-lives>.
