# Supplementary material for: Mobility changes following COVID-19 stay-at-home policies varied by socioeconomic measures: An observational study in Ontario, Canada
Source: PLOS Glob Public Health. 2024 Nov 26;4(11):e0002926. doi: 10.1371/journal.pgph.0002926 (PMC11594434; doi:10.1371/journal.pgph.0002926)
Supplement: S2 Table — (DOCX) [file pgph.0002926.s007.docx]

**S2 Table. Socioeconomic variables from Statistics Canada 2016 Census of Population**

| Measure (*Source*)^a^ | Definition of indicator | Notes^b^ |
| --- | --- | --- |
| Population size  (100% *of census sample*) | Total population count of a census tract (CT) |  |
| Income  (*100% of census sample*)^c^ | Quintile rank of a CT’s after-tax income per person equivalent, weighted by population | After-tax income is calculated for each household from the income for all household members. Calendar year 2015 is the reference period for all income variables in the 2016 Census. Single-person equivalent is used to account for households of different sizes. The ranking is calculated from CTs within the Greater Toronto Area. |
| % essential workers  (25% *of census sample*) | Numerator: Number of persons in the labor force who have occupations in one of the following categories: Manufacturing/utilities, Trades/transport/equipment operators, Sales/services, Resources/agriculture/production  Denominator: Total labor force population aged 15 years and over in private households in the CT | Occupations are assigned according to the National Occupancy Classification (2016). Occupation was chosen over “Industry” to better represent the type of work performed and skill-level required by a population rather than the industry that provides the employment. Numerators may be defined separately (“or”) or added together in different combination sets (“and”). “Labor Force” is all persons in private households aged 15 years and older who were either employed or unemployed during the week of Sunday, May 1 to Saturday, May 7, 2016. |

^a^“Sample” refers to the short-form Census questionnaire (*100%* sample) or to the long-form questionnaire, received by a random sample of households (*25%* sample). It is mandatory for recipients to respond to the questionnaires. Statistical inferences for the entire population are drawn from the subset of responses of the long-form questionnaire; these inferences are reported in the tabulated values provided by Statistics Canada. Note that income information was collected solely from administrative data sources (*100%* sample) and were not part of either questionnaire.

^b^Additional details about variable definitions may be included the Census Dictionary; please refer to Statistics Canada’s Dictionary for the 2016 Census of Population for complete definitions. Some definitions provided here are taken verbatim from source.

^c^Income quintiles for the City of Toronto / Toronto Public Health Unit were tabulated by ICES from data contained in PCCF+ (version 7B) and adjusted for population size. [1]

**References**

1. Postal Code Conversion File Plus (PCCF+) Version 7B, Reference Guide. November 2018 Postal codes. Statistics Canada2018.
