# Supplementary material for: Mobility changes following COVID-19 stay-at-home policies varied by socioeconomic measures: An observational study in Ontario, Canada
Source: PLOS Glob Public Health. 2024 Nov 26;4(11):e0002926. doi: 10.1371/journal.pgph.0002926 (PMC11594434; doi:10.1371/journal.pgph.0002926)
Supplement: S3 Table — (DOCX) [file pgph.0002926.s008.docx]

**S3 Table. Neighborhood-level^a^ socioeconomic characteristics across 1254 census tracts in the Greater Toronto Area^b^.**

| Quintiles (Q)^c^ | Total population^d^ | N^e^ | Median (range)^f^  Income^g^ [CAD] | Median (range)  % Essential worker^h^ |
| --- | --- | --- | --- | --- |
| Income |  |  |  |  |
| 1 (highest income) | 1,279,123 | 263 | 64,614 (58,829;135,145) | 0.31 (0.12; 0.46) |
| 2 | 1,277,104 | 236 | 55,688 (52,212; 58,808) | 0.39 (0.17; 0.56) |
| 3 | 1,279,041 | 253 | 48,478 (45,590; 52,141) | 0.43 (0.18; 0.61) |
| 4 | 1,274,503 | 246 | 41,933 (38,394; 45,565) | 0.46 (0.21; 0.71) |
| 5 (lowest income) | 1,274,013 | 256 | 34,118 (17,830; 38,367) | 0.52 (0.16; 0.72) |
| % Essential worker |  |  |  |  |
| 1 (lowest %) | 1,270,290 | 263 | 57,786 (23,953; 135,145) | 0.24 (0.12; 0.3) |
| 2 | 1,280,122 | 231 | 55,787 (30,551; 77,612) | 0.34 (0.3; 0.37) |
| 3 | 1,279,013 | 251 | 52,821 (21,513; 71,048) | 0.41 (0.37; 0.44) |
| 4 | 1,275,511 | 249 | 45,442 (18,716; 62,807) | 0.48 (0.44; 0.52) |
| 5 (highest %) | 1,278,848 | 260 | 37,625 (17,830; 57,274) | 0.57 (0.52; 0.72) |

The lowest-income neighborhoods (median income $34,118 CAD; range [$17,830-$38,367]) had the highest proportion of essential workers (median 52%, range [16%-72%]). Conversely, the highest income neighborhoods (median income $64,614 CAD, range [$58,829-$135,145]) overlapped with the lowest proportion essential workers (median 31%, range [12%-46%]).

^a^Neighborhood-level variables are at the level of census tract;

^b^Greater Toronto Area comprised of five public health units (Toronto, Peel, Halton, York, and Durham);

^c^Quintile (Q) was calculated across five public health units, weighted by neighborhood-level population in terms of the socioeconomic variables;

^d^Total population = total population of census tracts in each quintile;

^e^N = number of census tracts in each quintile;

^f^Median (range) = median (min, max);

^g^Income = after-tax income per-person equivalent in the household, aggregated at neighborhood level and given as a median and range for the quintile;

^h^% Essential worker = proportion of the working population engaged in essential services. Essential services include: trades, transport, and equipment operation; sales and services; manufacturing and utilities; and resources, agriculture, and production.
