# Supplementary material for: Mobility changes following COVID-19 stay-at-home policies varied by socioeconomic measures: An observational study in Ontario, Canada
Source: PLOS Glob Public Health. 2024 Nov 26;4(11):e0002926. doi: 10.1371/journal.pgph.0002926 (PMC11594434; doi:10.1371/journal.pgph.0002926)
Supplement: S4 Table — (DOCX) [file pgph.0002926.s009.docx]

**S4 Table. Device penetration in 1240 census tracts within the Greater Toronto Area^a^ stratified by neighborhood-level^b^ socioeconomic measures during the pre-restriction and post-restriction^c^ periods related to the two restrictions examined in the study.**

| Quintiles (Q)^d^ | N^e^ | Mean devices^f^ (SD^g^) | Mean population^h^ (SD) | Mean device coverage^i^ (SD) |
| --- | --- | --- | --- | --- |
| Income^j^ |  |  |  |  |
| 1 (highest) | 259 | 738 (634.53) | 4,858 (2,120.35) | 0.15 (0.11) |
| 2 | 229 | 843 (750.03) | 5,411 (2,511.18) | 0.16 (0.16) |
| 3 | 252 | 698 (672.49) | 5,073 (2,578.06) | 0.14 (0.16) |
| 4 | 245 | 601 (343.96) | 5,182 (1,700.93) | 0.12 (0.05) |
| 5 (lowest) | 255 | 496 (263.13) | 4,995 (1,758.38) | 0.10 (0.04) |
| % Essential worker^k^ |  |  |  |  |
| 1 (lowest %) | 261 | 542 (347.64) | 4,847 (2,347.85) | 0.11 (0.05) |
| 2 | 231 | 767 (555.34) | 5,542 (2,392.26) | 0.14 (0.08) |
| 3 | 241 | 796 (789.13) | 5,092 (2,069.41) | 0.15 (0.13) |
| 4 | 248 | 714 (633.51) | 5,124 (2,159.39) | 0.14 (0.13) |
| 5 (highest %) | 259 | 567 (416.09) | 4,925 (1,761.18) | 0.12 (0.14) |

^a^Greater Toronto Area comprised of five public health units (Toronto, Peel, Halton, York, and Durham);

^b^Neighborhood-level variables are at the level of census tract;

^c^Pre-restriction and post-restriction periods are three weeks before and after restriction implementation, respectively; and excluded the week of implementation;

^d^Quintile (Q) was calculated across five public health units, weighted by census tract population size in terms of the socioeconomic variables;

^e^N = number of census tract in each quintile;

^f^Mean devices = average daily number of unique devices in each census tract during study periods in each quintile;

^g^SD = standard deviation of measures in each quintile;

^h^Mean population = average population of census tracts in each quintile;

^f^SD (population) = standard deviation of census tract population in each quintile;

^i^Mean device coverage = average device coverage by census tracts (average daily number of unique devices in each census tract divided by the census tract population size) during study periods in each quintile;

^j^Income = after-tax income per person equivalent (CAD) in the household, aggregated at the level of the census tract and given as a median and range for the quintile;

^k^% Essential worker = proportion of the working population engaged in essential services. Essential services include: trades, transport, and equipment operation; sales and services; manufacturing and utilities; and resources, agriculture, and production.
