# Supplementary material for: Mobility changes following COVID-19 stay-at-home policies varied by socioeconomic measures: An observational study in Ontario, Canada
Source: PLOS Glob Public Health. 2024 Nov 26;4(11):e0002926. doi: 10.1371/journal.pgph.0002926 (PMC11594434; doi:10.1371/journal.pgph.0002926)
Supplement: S5 Table — (DOCX) [file pgph.0002926.s010.docx]

**S5 Table. Area-level^a^ difference-in-differences analysis of the second restriction with mixed-effect modeling in Greater Toronto Area^b^ (Model 1).**

| Covariate | Coefficient^c^ | Standard error | 95% CI^d^ |
| --- | --- | --- | --- |
| Intercept | -14.31 | 0.72 | (-15.98; -12.58) |
| Week^e^ 2 | 3.23 | 0.24 | (2.75; 3.7) |
| Week 3 | -0.05 | 0.24 | (-0.53; 0.42) |
| Week 4 | 2.65 | 0.31 | (2.05; 3.25) |
| Week 5 | 0.21 | 0.31 | (-0.39; 0.82) |
| Week 6 | 0.72 | 0.31 | (0.12; 1.33) |
| Group^f^ | -3.17 | 1.08 | (-5.82; -0.58) |
| Restriction^g^ | -0.96 | 0.29 | (-1.53; -0.38) |

^a^Area-level variable at the level of census tract;

^b^Greater Toronto Area comprised of five public health unit (Toronto, Peel, Halton, York, and Durham);

^c^Coefficients represent the estimated parameters for Equation (1) as detailed in S3 Text;

^d^95% CI = 95% confidence interval;

^e^Week *t*, *t* = 1, 2, .., 6, one of the six weeks during the study period, where Week 1 (i.e. *t* = 1) is the baseline level;

^f^Group is a time-invariant binary indicator denoting whether a census tract was in the treatment/intervention group (i.e. Toronto and Peel public health units);

^g^Restriction is a binary indicator denoting whether a census tract was under restriction in week *t*.
