# Supplementary material for: Mobility changes following COVID-19 stay-at-home policies varied by socioeconomic measures: An observational study in Ontario, Canada
Source: PLOS Glob Public Health. 2024 Nov 26;4(11):e0002926. doi: 10.1371/journal.pgph.0002926 (PMC11594434; doi:10.1371/journal.pgph.0002926)
Supplement: S6 Table — (DOCX) [file pgph.0002926.s011.docx]

**S6 Table. Difference-in-differences analysis of the second restriction with mixed-effect modeling in Greater Toronto Area^a^ by area-level^b^ income^c^ quintiles (Q)^d^ (Model 2A).**

| Covariate | Coefficient^e^ | Standard error | 95% CI^f^ |
| --- | --- | --- | --- |
| Intercept | -14.79 | 0.9 | (-16.74; -12.82) |
| Week^g^ 2 | 3.19 | 0.53 | (2.15; 4.22) |
| Week 3 | -0.77 | 0.53 | (-1.81; 0.26) |
| Week 4 | 2.4 | 0.6 | (1.23; 3.57) |
| Week 5 | -0.13 | 0.6 | (-1.3; 1.04) |
| Group^h^ | -3.26 | 1.32 | (-6.21; -0.32) |
| Income Q2 | 0.43 | 0.87 | (-1.28; 2.13) |
| Income Q3 | 1.23 | 0.95 | (-0.63; 3.09) |
| Income Q4 | 0.41 | 1.18 | (-1.91; 2.73) |
| Income Q5 (lowest) | 2.63 | 1.38 | (-0.08; 5.33) |
| Restriction^i^ | -2.71 | 0.61 | (-3.92; -1.51) |
| Week 2*Income Q2 | 0.08 | 0.77 | (-1.43; 1.59) |
| Week 2*Income Q3 | 0.38 | 0.75 | (-1.1; 1.86) |
| Week 2*Income Q4 | 0.18 | 0.76 | (-1.31; 1.66) |
| Week 2*Income Q5 | -0.42 | 0.75 | (-1.89; 1.05) |
| Week 3*Income Q2 | 0.41 | 0.77 | (-1.11; 1.92) |
| Week 3*Income Q3 | 0.63 | 0.75 | (-0.85; 2.1) |
| Week 3*Income Q4 | 1.43 | 0.76 | (-0.06; 2.91) |
| Week 3*Income Q5 | 1.14 | 0.75 | (-0.33; 2.61) |
| Week 4*Income Q2 | 0.55 | 0.86 | (-1.14; 2.24) |
| Week 4*Income Q3 | 0.8 | 0.9 | (-0.97; 2.58) |
| Week 4*Income Q4 | -0.36 | 1.07 | (-2.45; 1.73) |
| Week 4*Income Q5 | -0.96 | 1.2 | (-3.31; 1.39) |
| Week 5*Income Q2 | 0.38 | 0.86 | (-1.31; 2.07) |
| Week 5*Income Q3 | 1.21 | 0.9 | (-0.56; 2.98) |
| Week 5*Income Q4 | -1.06 | 1.07 | (-3.15; 1.03) |
| Week 5*Income Q5 | -0.08 | 1.2 | (-2.44; 2.27) |
| Week 6*Income Q2 | 0.48 | 0.86 | (-1.21; 2.17) |
| Week 6*Income Q3 | 1.32 | 0.9 | (-0.45; 3.09) |
| Week 6*Income Q4 | -0.52 | 1.07 | (-2.61; 1.58) |
| Week 6*Income Q5 | -0.18 | 1.2 | (-2.53; 2.17) |
| Group*Income Q2 | -0.29 | 1.14 | (-2.52; 1.94) |
| Group*Income Q3 | -1.21 | 1.13 | (-3.43; 1.01) |
| Group*Income Q4 | -0.35 | 1.31 | (-2.92; 2.23) |
| Group*Income Q5 | -0.5 | 1.49 | (-3.42; 2.41) |
| Restriction*Income^j^ Q2 | 1.22 | 0.9 | (-0.55; 2.99) |
| Restriction*Income Q3 | 0.81 | 0.89 | (-0.94; 2.56) |
| Restriction*Income Q4 | 3.61 | 1.04 | (1.57; 5.66) |
| Restriction*Income Q5 | 3.28 | 1.17 | (0.98; 5.58) |

^a^Greater Toronto Area comprised of five public health unit (Toronto, Peel, Halton, York, and Durham);

^b^Area-level variables at the level of census tract;

^c^Income = after-tax income per person equivalent (CAD) in the household, aggregated at the level of the census tract;

^d^Quintile (Q) was calculated across five public health units, weighted by census tract population size in terms of the socioeconomic variables, and Q1 is the baseline level which refers to the highest income group;

^e^Coefficients represent the estimated parameters for Equation (2) as detailed in S3 Text;

^f^95% CI = 95% confidence interval;

^g^Week *t*, *t* = 1, 2, .., 6, one of the six weeks during the study period, where Week 1 (i.e. *t* = 1) is the baseline level;

^h^Group is a time-invariant binary indicator denoting whether a census tract was in the treatment/intervention group (i.e. Toronto and Peel public health units);

^i^Restriction is a binary indicator denoting whether a census tract was under restriction in week *t*;

^j^Restiction*Income is the effect modification on the restriction effect by income quintiles. We conducted an F-test with numerator degrees of freedom equal to 4 (p<0.05).
