# Supplementary material for: Mobility changes following COVID-19 stay-at-home policies varied by socioeconomic measures: An observational study in Ontario, Canada
Source: PLOS Glob Public Health. 2024 Nov 26;4(11):e0002926. doi: 10.1371/journal.pgph.0002926 (PMC11594434; doi:10.1371/journal.pgph.0002926)
Supplement: S7 Table — (DOCX) [file pgph.0002926.s012.docx]

**S7 Table. Difference-in-differences analysis of the second restriction with mixed-effect modeling in Greater Toronto Area^a^ by area-level^b^ essential worker^c^ quintiles (Q)^d^ (Model 2B).**

| Covariate | Coefficient^e^ | Standard error | 95% CI^f^ |
| --- | --- | --- | --- |
| Intercept | -16.88 | 1 | (-18.86; -14.81) |
| Week^g^ 2 | 3.91 | 0.53 | (2.88; 4.94) |
| Week 3 | -1.44 | 0.53 | (-2.48; -0.41) |
| Week 4 | 1.15 | 0.77 | (-0.35; 2.66) |
| Week 5 | -0.58 | 0.77 | (-2.08; 0.93) |
| Group^h^ | -3.81 | 1.24 | (-6.55; -1.25) |
| Essential worker Q2 | 0.82 | 1.02 | (-1.19; 2.83) |
| Essential worker Q3 | 2.38 | 1.04 | (0.34; 4.42) |
| Essential worker Q4 | 5.16 | 1.1 | (3; 7.33) |
| Essential worker Q5 (highest) | 5.58 | 1.27 | (3.08; 8.07) |
| Restriction^i^ | -1.43 | 0.72 | (-2.85; -0.01) |
| Week 2*Essential worker Q2 | -0.41 | 0.77 | (-1.91; 1.09) |
| Week 2*Essential worker Q3 | -0.42 | 0.76 | (-1.9; 1.07) |
| Week 2*Essential worker Q4 | -0.6 | 0.75 | (-2.07; 0.88) |
| Week 2*Essential worker Q5 | -1.93 | 0.74 | (-3.39; -0.48) |
| Week 3*Essential worker Q2 | 1 | 0.77 | (-0.5; 2.5) |
| Week 3*Essential worker Q3 | 1.67 | 0.76 | (0.19; 3.16) |
| Week 3*Essential worker Q4 | 3.13 | 0.75 | (1.66; 4.61) |
| Week 3*Essential worker Q5 | 1.2 | 0.74 | (-0.26; 2.66) |
| Week 4*Essential worker Q2 | 1.81 | 0.99 | (-0.13; 3.76) |
| Week 4*Essential worker Q3 | 2.17 | 0.99 | (0.23; 4.11) |
| Week 4*Essential worker Q4 | 1.69 | 1.04 | (-0.34; 3.72) |
| Week 4*Essential worker Q5 | 1.68 | 1.15 | (-0.58; 3.93) |
| Week 5*Essential worker Q2 | 0.86 | 0.99 | (-1.09; 2.81) |
| Week 5*Essential worker Q3 | 1.24 | 0.99 | (-0.7; 3.18) |
| Week 5*Essential worker Q4 | 0.64 | 1.04 | (-1.39; 2.67) |
| Week 5*Essential worker Q5 | 0.98 | 1.15 | (-1.27; 3.23) |
| Week 6*Essential worker Q2 | 1.38 | 0.99 | (-0.57; 3.33) |
| Week 6*Essential worker Q3 | 2.57 | 0.99 | (0.63; 4.52) |
| Week 6*Essential worker Q4 | 1.27 | 1.04 | (-0.76; 3.3) |
| Week 6*Essential worker Q5 | 2.35 | 1.15 | (0.1; 4.6) |
| Group*Essential worker Q2 | 0.85 | 1.16 | (-1.43; 3.13) |
| Group*Essential worker Q3 | 1.15 | 1.17 | (-1.16; 3.45) |
| Group*Essential worker Q4 | -1.92 | 1.19 | (-4.26; 0.42) |
| Group*Essential worker Q5 | 0.14 | 1.33 | (-2.47; 2.76) |
| Restriction*Essential worker^j^ Q2 | 0.85 | 0.97 | (-1.05; 2.76) |
| Restriction*Essential worker Q3 | -1.04 | 0.96 | (-2.93; 0.84) |
| Restriction*Essential worker Q4 | 1.9 | 0.98 | (-0.02; 3.82) |
| Restriction*Essential worker Q5 | 0.76 | 1.09 | (-1.37; 2.89) |

^a^Greater Toronto Area comprised of five public health unit (Toronto, Peel, Halton, York, and Durham);

^b^Area-level variables at the level of census tract;

^c^Essential worker = proportion of the working population engaged in essential services. Essential services include: trades, transport, and equipment operation; sales and services; manufacturing and utilities; and resources, agriculture, and production;

^d^Quintile (Q) was calculated across five public health units, weighted by census tract population size in terms of the socioeconomic variables, and Q1 is the baseline level which refers to the lowest proportion of essential workers;

^e^Coefficients represent the estimated parameters for Equation (2) as detailed in S3 Text;

^f^95% CI = 95% confidence interval;

^g^Week *t*, *t* = 1, 2, .., 6, one of the six weeks during the study period, where Week 1 (i.e. *t* = 1) is the baseline level;

^h^Group is a time-invariant binary indicator denoting whether a census tract was in the treatment/intervention group (i.e. Toronto and Peel public health units);

^i^Restriction is a binary indicator denoting whether a census tract was under restriction in week *t*.

^j^Restiction*Essential worker is the effect modification on the restriction effect by essential worker quintiles. We conducted an F-test with numerator degrees of freedom equal to 4 (p<0.05).
