# Supplementary material for: Mobility changes following COVID-19 stay-at-home policies varied by socioeconomic measures: An observational study in Ontario, Canada
Source: PLOS Glob Public Health. 2024 Nov 26;4(11):e0002926. doi: 10.1371/journal.pgph.0002926 (PMC11594434; doi:10.1371/journal.pgph.0002926)
Supplement: S8 Table — (DOCX) [file pgph.0002926.s013.docx]

**S8 Table. Difference-in-differences analysis of the second restriction with mixed-effect modeling in Greater Toronto Area^a^ by area-level^b^ socioeconomic measures (Model 2A and Model 2B).**

| Quintiles (Q)^c^ | Adjusted mobility^d^ change^e^ | 95% CI^f^ |
| --- | --- | --- |
| Income^g^ |  |  |
| 1 (highest) | -2.71 | (-3.92; -1.51) |
| 2 | -1.5 | (-3.15; 0.16) |
| 3 | -1.91 | (-3.52; -0.29) |
| 4 | 0.9 | (-1.19; 3.00) |
| 5 (lowest) | 0.56 | (-1.93; 3.05) |
| % Essential workers^h^ |  |  |
| 1 (lowest) | -1.43 | (-2.85; -0.01) |
| 2 | -0.58 | (-2.19; 1.03) |
| 3 | -2.48 | (-4.05; -0.90) |
| 4 | 0.47 | (-1.17; 2.11) |
| 5 (highest) | -0.67 | (-2.68; 1.35) |

^a^Greater Toronto Area comprised of five public health unit (Toronto, Peel, Halton, York, and Durham);

^b^Area-level variables at the level of census tract;

^c^Quintile (Q) was calculated across five public health units, weighted by census tract population size in terms of the socioeconomic variables;

^d^Adjusted mobililty = crude mobility in 2020 minus crude mobility in 2019;

^e^Adjusted mobility change refers to the estimates of the adjusted mobility change by socioeconomic quintiles, following the second restriction. The estimates were calculated by adding the effect modification on the second restriction effect by socioeconomic quintiles to the estimated effect of the restriction;

^f^95% CI = 95% confidence interval;

^g^Income = after-tax income per person equivalent (CAD) in the household, aggregated at the level of the census tract;

^h^% Essential worker = proportion of the working population engaged in essential services. Essential services include: trades, transport, and equipment operation; sales and services; manufacturing and utilities; and resources, agriculture, and production.
