# Supplementary material for: Mobility changes following COVID-19 stay-at-home policies varied by socioeconomic measures: An observational study in Ontario, Canada
Source: PLOS Glob Public Health. 2024 Nov 26;4(11):e0002926. doi: 10.1371/journal.pgph.0002926 (PMC11594434; doi:10.1371/journal.pgph.0002926)
Supplement: S1 Fig — (DOCX) [file pgph.0002926.s014.docx]

**
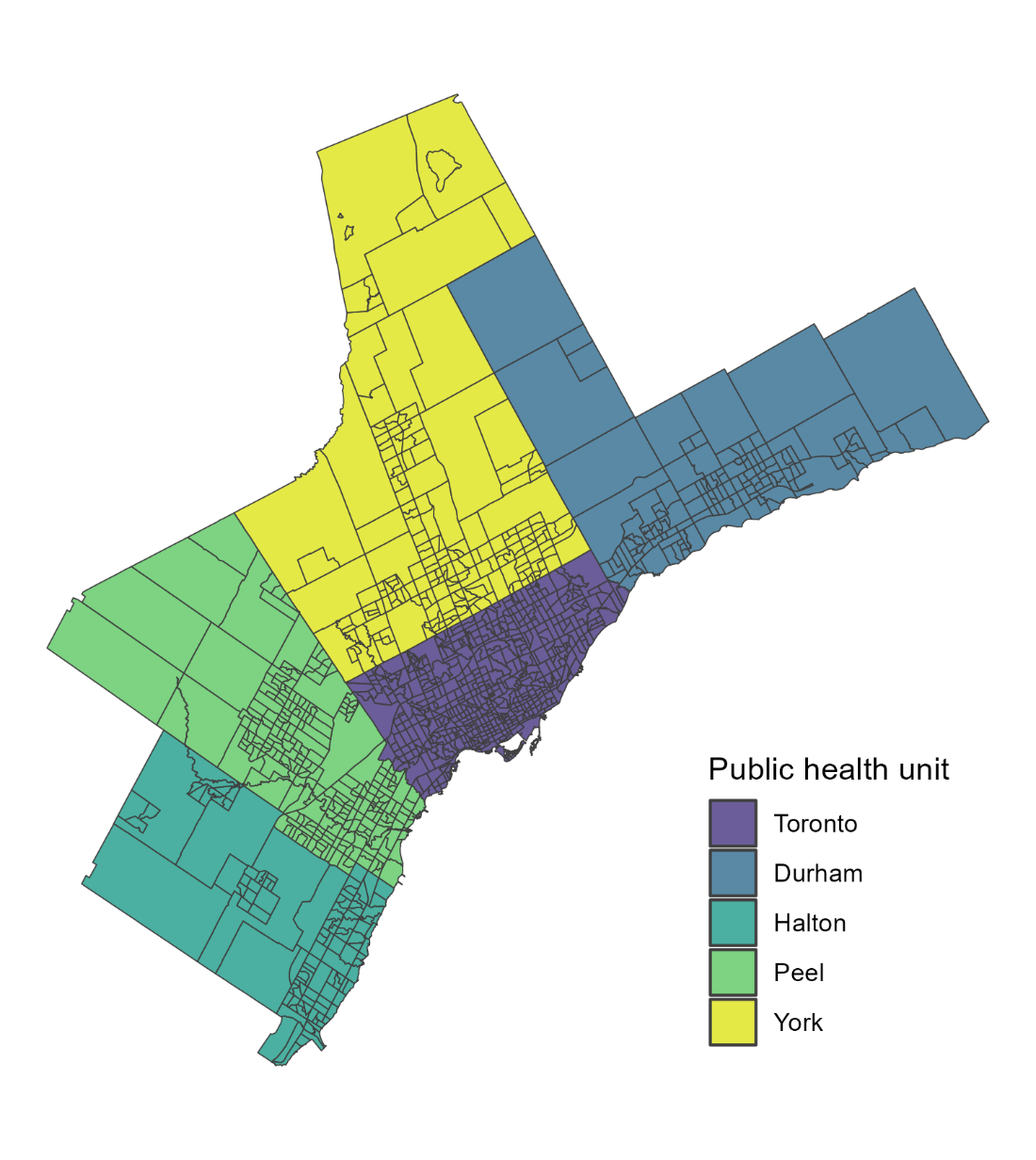
**

**S1 Fig. Five public health units consist of census tracts within the Greater Toronto Area.** Map generated by R using public raw data at the census-tract level from Statistics Canada 2016 Census - Boundary file. (<https://www12.statcan.gc.ca/census-recensement/2011/geo/bound-limit/bound-limit-2016-eng.cfm>). No external base layer or proprietary shapefiles were used.
