# Supplementary material for: Mobility changes following COVID-19 stay-at-home policies varied by socioeconomic measures: An observational study in Ontario, Canada
Source: PLOS Glob Public Health. 2024 Nov 26;4(11):e0002926. doi: 10.1371/journal.pgph.0002926 (PMC11594434; doi:10.1371/journal.pgph.0002926)
Supplement: S2 Fig — (DOCX) [file pgph.0002926.s015.docx]

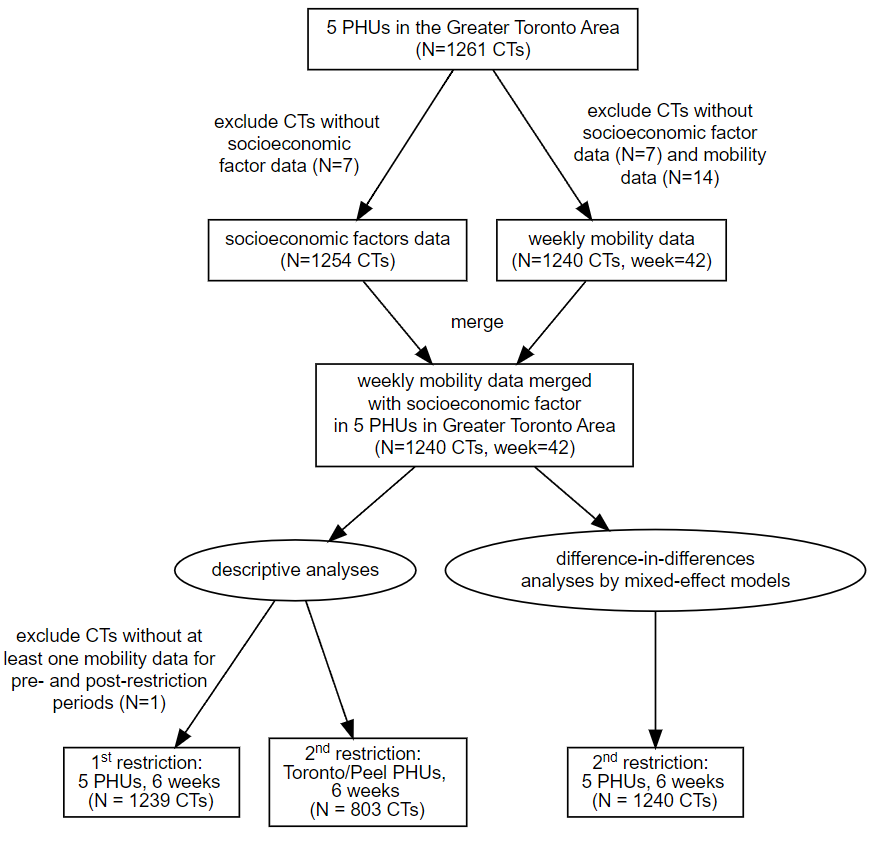


**S2 Fig. Flow diagram for data process and for descriptive analyses and difference-in-differences analyses by mixed-effect model.** We have 1261 census tracts (CTs) across all five public health units (PHU) in the Greater Toronto Area. They are Toronto, Peel, York, Durham and Halton PHUs. N is the corresponding number of CTs we excluded or in the dataset. We defined pre- and post-restriction periods as three weeks before and after restriction implementation, respectively; and excluded the week of implementation. In total, we had six weeks data for each analysis. For each analysis, we only kept the census tracts with at least one mobility data for each pre-restriction and post-restriction period. Thus, we excluded 1 census tract for descriptive analysis of the first restriction. The 7 neighborhoods with missing data on socioeconomic variables represented populations of 0 to 149. The 14 neighborhoods without mobility data ranged in population size from 261 to 9,492. Compared to neighborhoods with mobility data, the 14 neighborhoods without mobility data had a lower median population size (median 490 versus 4,648) and a higher median income ($55,269 CAD vs $48,333 CAD). The median essential workers proportion is similar between neighborhoods with or without mobility data.
