# Supplementary material for: Mobility changes following COVID-19 stay-at-home policies varied by socioeconomic measures: An observational study in Ontario, Canada
Source: PLOS Glob Public Health. 2024 Nov 26;4(11):e0002926. doi: 10.1371/journal.pgph.0002926 (PMC11594434; doi:10.1371/journal.pgph.0002926)
Supplement: S3 Fig — (DOCX) [file pgph.0002926.s016.docx]

**
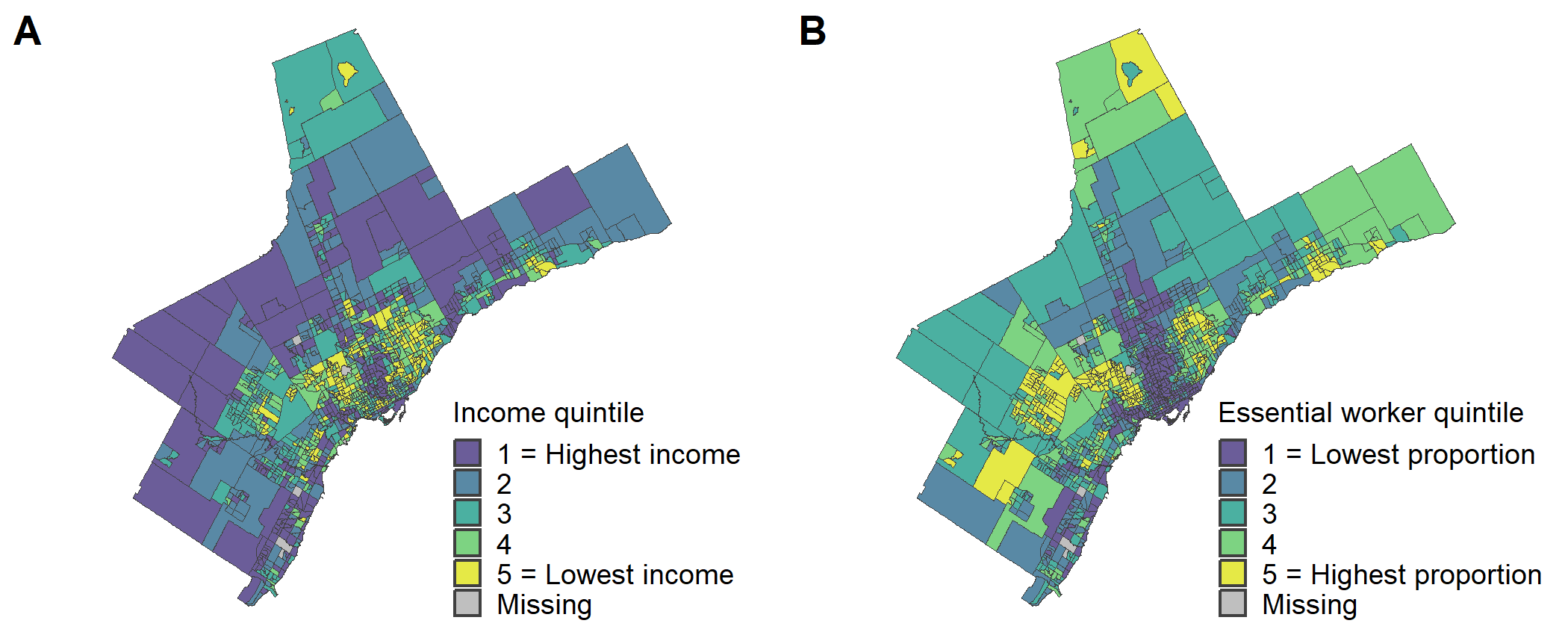
**

**S3 Fig. The geographic distribution of income quintiles and essential worker quintiles at the census-tract level in five public health units within the Greater Toronto Area.** Income reflects the per-person equivalent income in the household. Essential services include: trades, transport, and equipment operation; sales and services; manufacturing and utilities; and resources, agriculture, and production. Quintiles (Q) are weighted by census tract level population. Census tracts are Statistics Canada geographic units that are only used within metropolitan, with relative small population sizes. Greater Toronto Area comprised of five public health units (Toronto, Peel, Halton, York, and Durham). Map generated by R using public raw data at the census-tract level from Statistics Canada 2016 Census - Boundary file. (<https://www12.statcan.gc.ca/census-recensement/2011/geo/bound-limit/bound-limit-2016-eng.cfm>). No external base layer or proprietary shapefiles were used.
