# Supplementary material for: Mobility changes following COVID-19 stay-at-home policies varied by socioeconomic measures: An observational study in Ontario, Canada
Source: PLOS Glob Public Health. 2024 Nov 26;4(11):e0002926. doi: 10.1371/journal.pgph.0002926 (PMC11594434; doi:10.1371/journal.pgph.0002926)
Supplement: S4 Fig — (DOCX) [file pgph.0002926.s017.docx]

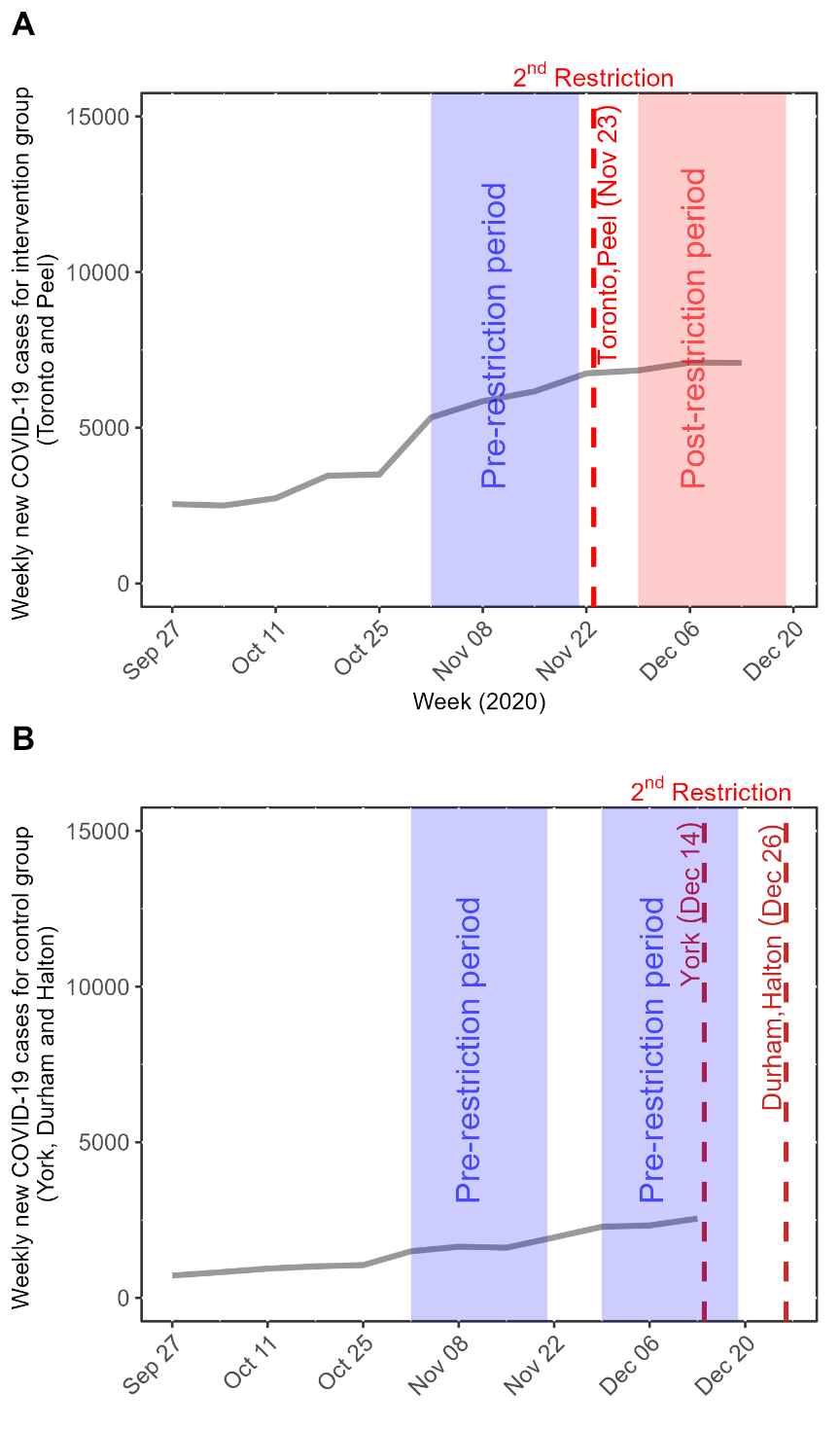


**S4 Fig. Epidemic curve and the restriction policy timing for the intervention group (Toronto and Peel) and the control group (York, Durham and Halton) in Greater Toronto Area**. The 2^nd^ restriction enacted on November 23, 2020 in Toronto and Peel public health units and in the remaining public health units on December 14 or 26, 2023 within the Greater Toronto Area. Panel A depicts the restriction policy timing for the intervention group (Toronto and Peel public health units) that had the 2^nd^ restriction on November 23, 2020. Panel B depicts the restriction policy timing for the control group (York, Durham and Halton) that without restriction policy on November 23, 2020. The vertical dashed lines depict the timelines for the 2^nd^ restriction policy across five public health units. Each shaded area includes three-week period. The shaded areas in blue represent the periods before the restriction used in the analysis, and the shaded areas in red represent the periods after the restriction used in the analysis. We excluded the weeks that the 2^nd^ restriction policy first was enacted (on November 23, 2020) from the difference-in-differences analyses. The study period of difference-in-differences analyses covered all shaded areas. For the remaining three public health units (York, Durham and Halton), we defined the weeks that the 2^nd^ restriction policy was enacted as the period before restriction. The weekly cases comprise diagnosed cases, and exclude cases among residents of long-term care homes.
