# Supplementary material for: Mobility changes following COVID-19 stay-at-home policies varied by socioeconomic measures: An observational study in Ontario, Canada
Source: PLOS Glob Public Health. 2024 Nov 26;4(11):e0002926. doi: 10.1371/journal.pgph.0002926 (PMC11594434; doi:10.1371/journal.pgph.0002926)
Supplement: S5 Fig — (DOCX) [file pgph.0002926.s018.docx]

**
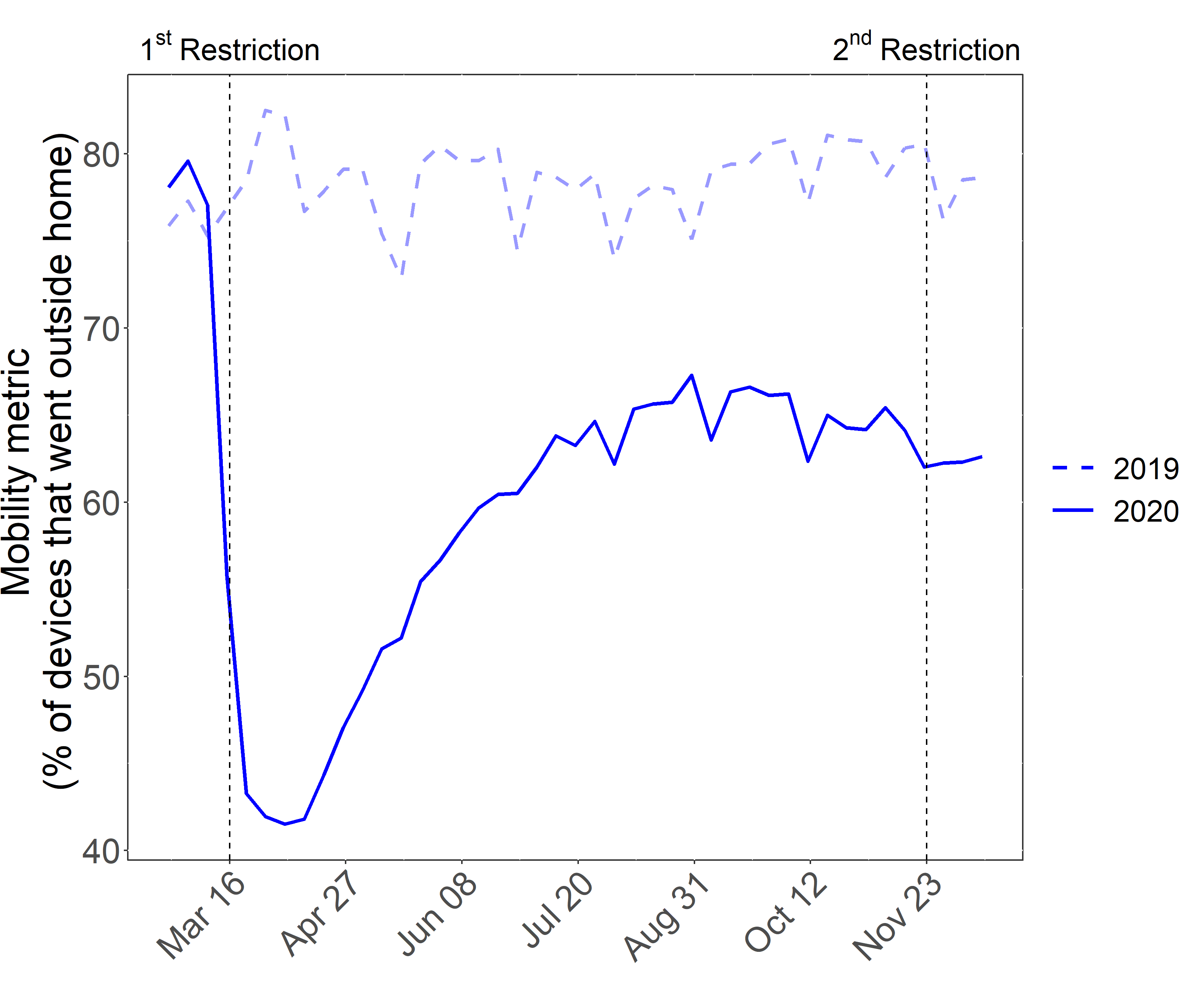
**

**S5 Fig. Mobility trajectories for 2019 and 2020 with the timing of two restrictions in the Greater Toronto Area.** Mobility metric is defined as average percent of devices that went outside home. The horizontal dashed line represents 2019 mobility as pre-pandemic mobility reference, while the solid lines represent 2020 mobility data. The vertical dashed lines depict the two COVID-19 restriction policies under examination: the 1^st^ restriction enacted March 17, 2020 across all five public health units (Toronto, Peel, Halton, York, and Durham) in the Greater Toronto Area; and the 2^nd^ restriction enacted November 23, 2020 in Toronto and Peel public health units within the Greater Toronto Area.
