# Supplementary material for: Mobility changes following COVID-19 stay-at-home policies varied by socioeconomic measures: An observational study in Ontario, Canada
Source: PLOS Glob Public Health. 2024 Nov 26;4(11):e0002926. doi: 10.1371/journal.pgph.0002926 (PMC11594434; doi:10.1371/journal.pgph.0002926)
Supplement: S6 Fig — (DOCX) [file pgph.0002926.s019.docx]

**
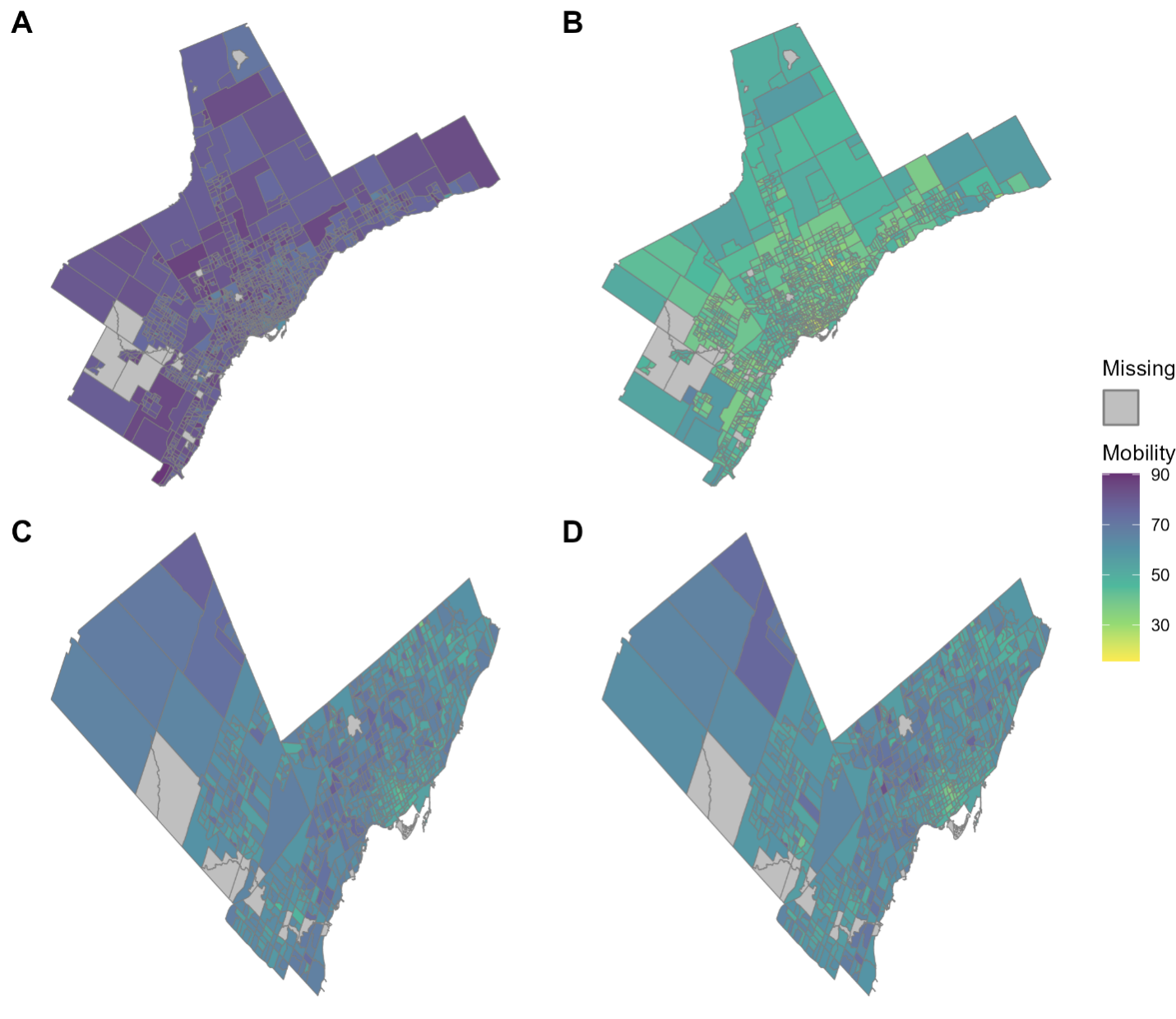
**

**S6 Fig. Maps for the three-week average mobility before and after 1^st^ restriction in five public health units within the Greater Toronto Area, and three-week average mobility before and after 2^nd^ restriction in Toronto and Peel public health units.** Panel A and B depict three-week average mobility (% devices that went outside home) at census tract level before (Panel A) and after (Panel B) 1^st^ restriction across all five public health units (Toronto, Peel, Halton, York, and Durham) in the Greater Toronto Area. Panel C and D depict three-week average mobility at census tract level before (Panel C) and after (Panel D) 2^nd^ restriction in Toronto and Peel public health unit only. Map generated by R using public raw data at the census-tract level from Statistics Canada 2016 Census - Boundary file. (<https://www12.statcan.gc.ca/census-recensement/2011/geo/bound-limit/bound-limit-2016-eng.cfm>). No external base layer or proprietary shapefiles were used.
